# Advances in Chemical and Biological Methods to Identify Microorganisms—From Past to Present

**DOI:** 10.3390/microorganisms7050130

**Published:** 2019-05-13

**Authors:** Ricardo Franco-Duarte, Lucia Černáková, Snehal Kadam, Karishma S. Kaushik, Bahare Salehi, Antonio Bevilacqua, Maria Rosaria Corbo, Hubert Antolak, Katarzyna Dybka-Stępień, Martyna Leszczewicz, Saulo Relison Tintino, Veruska Cintia Alexandrino de Souza, Javad Sharifi-Rad, Henrique Douglas Melo Coutinho, Natália Martins, Célia F. Rodrigues

**Affiliations:** 1 CBMA (Centre of Molecular and Environmental Biology), Department of Biology, University of Minho, 4710-057 Braga, Portugal; ricardofrancoduarte@gmail.com; 2 Institute of Science and Innovation for Bio-Sustainability (IB-S), University of Minho, 4710-057 Braga, Portugal; 3 Department of Microbiology and Virology, Faculty of Natural Sciences, Comenius University in Bratislava, Ilkovičova 6, 842 15 Bratislava, Slovakia; lucia.cernakova@uniba.sk; 4 Ramalingaswami Re-entry Fellowship, Department of Biotechnology, Government of India, Pune 411045, India; snehalgkad@gmail.com (S.K.); karishmaskaushik@gmail.com (K.S.K.); 5 Student Research Committee, School of Medicine, Bam University of Medical Sciences, Bam 14665-354, Iran; 6 Department of the Science of Agriculture, Food and Environment, University of Foggia, 71121 Foggia, Italy; antonio.bevilacqua@unifg.it (A.B.); mariarosaria.corbo@unifg.it (M.R.C.); 7 Institute of Fermentation Technology and Microbiology, Department of Biotechnology and Food Science, Lodz University of Technology, Wolczanska 171/173, 90-924 Lodz, Poland; hubert.antolak@p.lodz.pl (H.A.); katarzyna.dybka@p.lodz.pl (K.D.-S.); 8 Laboratory of Industrial Biotechnology, Bionanopark Ltd., Dubois 114/116, 93-465 Lodz, Poland; m.leszczewicz@bionanopark.pl; 9 Laboratory of Microbiology and Molecular Biology (LMBM), Department of Biological Chemistry/CCBS/URCA, Crato 63105-000, Brazil; saulorelison@gmail.com; 10 Departamento of Microbiology, Instituto Aggeu Magalhães/Fiocruz, Recife 50670-420, Brazil; veruska.cintia02@hotmail.com; 11 Zabol Medicinal Plants Research Center, Zabol University of Medical Sciences, Zabol 61615-585, Iran; 12 Faculty of Medicine, University of Porto, Alameda Professor Hernâni Monteiro, 4200-319 Porto, Portugal; ncmartins@med.up.pt; 13 Institute for Research and Innovation in Health (i3S), University of Porto, 4200-135 Porto, Portugal; 14 LEPABE–Dep. of Chemical Engineering, Faculty of Engineering, University of Porto, Rua Dr. Roberto Frias, s/n, 4200-465 Porto, Portugal

**Keywords:** identification methods, PCR, real-time PCR, RAPD PCR, MALDI-TOF, microscopy techniques, chromogenic media, DNA fingerprinting, WGS

## Abstract

Fast detection and identification of microorganisms is a challenging and significant feature from industry to medicine. Standard approaches are known to be very time-consuming and labor-intensive (e.g., culture media and biochemical tests). Conversely, screening techniques demand a quick and low-cost grouping of bacterial/fungal isolates and current analysis call for broad reports of microorganisms, involving the application of molecular techniques (e.g., 16S ribosomal RNA gene sequencing based on polymerase chain reaction). The goal of this review is to present the past and the present methods of detection and identification of microorganisms, and to discuss their advantages and their limitations.

## 1. Introduction

Microorganisms have always been extremely important for human life and bacteria, yeasts and molds have been known for both positive and negative reasons. Just like in the past, as it is now, they are inevitably associated with biotechnology, food sciences, medicine, genetic engineering, and other fields of life. On one hand, they are used for their unique features which enable the production of antibiotics, hormones, amino acids, and other therapeutic compounds, and also production of food and food-related products, as well as decomposition of components such as lignocellulosic biomass for second-generation ethanol or biogas [1]. At the same time, selected genetic features and biochemical abilities of microorganisms make them dangerous for industry (food spoilage) as well as human health [2]. In fact, it is estimated that approximately 1400 pathogens can cause human diseases. Pathogenic bacteria alone are etiological agents of 350 million cases of foodborne diseases [3]. In the United States alone, 48 million foodborne illnesses occur annually, which led to approximately 128,000 hospitalizations and 3000 deaths. Poor water hygiene can be a cause of 1.7 million deaths a year worldwide, and nine of 10 deaths are in children and virtually all of the deaths are in developing countries. Furthermore, the majority of the pathogens responsible for these diseases and deaths includes *Campylobacter jejuni*, enterotoxigenic *Escherichia coli*, *Shigella* spp. and *Vibrio cholerae*, *Aeromonas* spp., enterotoxigenic *Bacteroides fragilis*, *Clostridium difficile* as well as *Cryptosporidium parvum* [4].

Two factors determine the potential use of microorganisms in biotechnological processes, and also the pathogenicity of other strains are their genetic features and biochemical abilities. In the near future, industrial application as well as treatment of infection, will be possible after characterization, identification, and following taxonomic classification of the biological material. It is necessary to emphasize that taxonomy and systematics, very often used interchangeably, are in fact two different terms. Although systematics deals with the diversity of organisms, relationships, and possible interactions, taxonomy is a classification of organisms in a hierarchical structure of homogeneous groups that consist of descendants of the nearest common ancestor. Despite a high degree of phenotypic similarity, every assemblage of an individual shows some degree of phenotypic diversity due to genotypic variation. The greater the differences at the genetic level, the farther the related organisms are [1]. Commonly known and used examples of hierarchical classification are the kingdom, division, class, family, genus, species, and finally, strain. Research works in the field of classification, systematics, and identification of microorganisms are interconnected and have an impact on each other. Accurate identification affects taxonomic classification of microorganisms as well as their systematics, and vice versa. Therefore, the broader the research aimed at the characterization of an individual microorganism, the more precise its identification, and thus the classification and systematics [1]. Accordingly, the “polyphasic” methodology is centered on morphological and biochemical data complemented with molecular techniques data. The combination of the classical approach together with 16S rRNA genes, molecular fingerprinting techniques, and/or other molecular markers is considered an extremely important foundation for the identification and classification of microbes [2].

The accurate identification of microbes is essential for scientists involved in many areas of applied research and industry which ranges from clinical microbiology to food production. There are many criteria for the division of the abundance of methods used in the area of the identification of microorganism, however, generally they can be assigned to direct and indirect techniques (Table 1).

**Figure 1:**
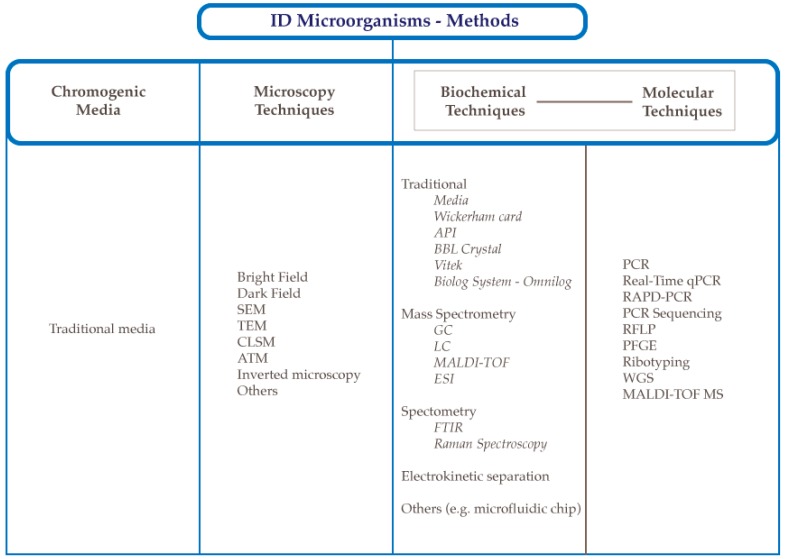
Methods for microorganism identification: chromogenic media and microscopy, biochemical and molecular techniques.

The time needed for microorganism identification based on the traditional approach which includes morphology, physiology, chemistry, and biochemical characterization is estimated to be at least 2 to 5 days, or even up to a dozen days in the case of molds. In addition, most phenotypic methods used in the microbiological laboratories are labor intensive as well as material consuming. Importantly, phenotypic methods are not always useful to identify unambiguously the microorganism to the species level, or much more often to the strain level [5]. One of the strategies to reduce time for microbial identification is the use of molecular biology techniques which may also be supplemented with numerous molecular fingerprinting techniques [6]. Each method has its strengths and weaknesses, and the most recent research approach involves the use of a compilation of multivariate techniques. Such implementation seems to have great potential for the future. In order to obtain the most precise identification, classification, and systematics of microorganisms, it is extremely important to choose appropriate techniques, as well as have a thorough understanding of the mechanisms of their action. Therefore, the purpose of this literature review is to provide a description of the traditional and novel methods of identification (Figure 1), as well as their strengths and limitations.

## 2. Methods to Identify Microorganisms

It is not possible to focus on microorganism identification without a reference to taxonomy, as it is a common idea that “identification is a part of taxonomy”. The word taxonomy comes from the Greek words taxis (arrangement) and nomos (law) and it is the science of the description, classification, and inventory of life [7,8,9]. Taxonomy dates back to ancient Greece, when Aristotle proposed the first classification of living organisms, and modern taxonomy was created by Linnaeus, who introduced the binomial classification which is still used today [10,11] and has been most responsible for the most recent taxonomy classifications [1].

### 2.1. Historical Evolution of Microorganism Identification

During the last decade, scientists have searched for the more prompt and effective means of microbial identification [1]. For several years, the phenotypic classification was the only identification approach, although this methodology always resulted in uncertainties and difficulties with the analysis. In fact, many reports described inaccuracies in phenotype-based speciation of microbial strains [12]. For example, Yeung et al. [13] described some typing discrepancies occurring in *Lactobacillus acidophilus* and *Lactobacillus casei* groups using phenotype-based techniques. Lagier et al. [14] reported some key experiments for a phenotype-based identification, generally used in the past, such as pH-based reactions, enzyme profile, carbon source utilization, acid detection, and cellular fatty acids. Generally, phenotypic methods were based on dichotomic keys, and the first test to be performed was Gram staining, followed by catalase and oxidase tests. First, carbohydrate metabolism was evaluated through acid production analysis (pH), and the end products (CO_2_, acetate, and lactate). Later, tests for enzymes began (glucuronidase, glucosidase, galactosidase, and fucosidase) [14]. An upgrade of the phenotype-based tests were the tests for protein and amino acid metabolism, such as the production of indole and H_2_S, gelatin and casein digestion, decarboxylation of lysine, ornithine, arginine, arginine dihydrolase, phenylalanine deaminase, and urease. Other phenotypic tests included those for lipid metabolism, such as lipase and lecithinase on egg yolk agar and the digestion of tween, or the experiments based on cell wall receptors, including optochin, lysozyme susceptibility, and bile solubility tests, frequently used for the differentiation of Gram-positive cocci [14]. Nowadays, numerous phenotypic characteristics can be concurrently verified in 4 to 48 h by applying commercial kits and/or automated phenotypic systems. Phenotypic tests are generally organized in dichotomous keys that are a series of yes/no tests to identify a microorganism. The tests are put in a logical order and each result indicates the next test to be done as first described by Skerman (1959) [15]. However, in 1965, Steel wrote “Difficulties in interpretation of keys arise where strains behave inconsistently in some respect…. Some characters are almost invariably positive or negative, but characters of such constancy are usually shared by similar organisms, and although they are important in characterizing an organism, have little value in distinguishing it from its neighbors.” [16]. Therefore, he realized that some difficulties could arise and lead to a misleading identification.

Therefore, in the late 20th century, molecular tools and sequence databases emerged. These methods, significantly contributed to expand the power of microbiology and increased the number of known microbial species (1971 in 1980, 8168 in 2007, and 12,391 in 2013) [14]. According to Madigan et al. [17], the date of birth of molecular biology was 1941, when DNA was discovered as the genetic material (although the bacterial genetics was discovered later, in 1946). The use of genetics as a tool for bacterial identification started in 1985 with the design of PCR (polymerase chain reaction). Since then, many methods have been developed and designed (culture dependent and culture independent approaches), based on different principles (DNA sequencing, 16S rRNA sequencing, and hybridization). Omics tools, metagenomics, proteomics, lipidomics, transcriptomics, and metabolomics [18], aim at a collective high-throughput characterization and quantification of pools of biological molecules that translate into the structure, function, and dynamics of an organism or organisms [19]. These approaches have some strong practical implications and are currently used for many purposes (e.g., microbial ecology, phylogeny, functional genome analysis, and transcriptional profiling) [18]. The most recent advances of microbial identification occurred in the omics era and represent something similar to a rediscovery of past approaches and methods. It is culturomics and represents a rebirth of some phenotypic approaches. It was first proposed for probiotics by Donelli et al. [12] and it was based on the polyphasic approach proposed by Colwell [20] for the *Vibrio* genus. In this approach, even if phenotypic characters frequently meet between genetically dissimilar species, molecular methods alone are frequently not able to fix clear limits among phylogenetically associated species [12]. Accordingly, a restructured scheme for the correct identification must focus on its morphological, physiological and biochemical features, and its genetic profile [12].

### 2.2. Identification Methods Using Chromogenic Media

In these methods, the identification of microorganisms based on cultivation has the initial objective of obtaining pure culture. A pure culture containing a single type of microorganism can be obtained in various ways from enrichment cultures. 

Frequently used isolation methods include seeding by dewatering, deep seeding in solid media, and liquid dilution. For organisms that form colonies in solid medium plates, the technique of seeding by exhaustion is fast, easy, and the method of choice. From the repeated collection and seeding of an isolated colony, a pure culture can be obtained. By using appropriate incubation devices it is possible to purify both aerobic and anaerobic organisms in solid medium plates by using the seeding method by depletion. It is also important to point out that through cultivation, the microorganism can be identified from the production of certain metabolites released in the medium with lactic acid and others, and therefore the technique is based on the reaction of the medium with the released metabolites [21]. On the other hand, the identification of microorganisms that is based on cultivation alone is quite incomplete, since the enrichment distortion is possibly a serious problem in studies on biodiversity dependent on cultivation. In fact, microbial ecologists estimate that, to date, less than 0.1% of the phylogotypes revealed by community molecular analysis exist in the form of laboratory cultures [17].

### 2.3. Microscopy Techniques

The microscope is an essential identification tool for microorganisms present in a natural sample. Microscopy images enable analysis of shape, tracking of motion, and classification of biological objects. The microscope-based observation is still frequently applied to define the morphological differences of interesting bacteria, such as streptococci, staphylococci, bacilli (e.g., *Listeria monocytogenes*, *E. coli* or *Salmonella* spp.), and *Vibrio*, in both clinical and research sceneries [22,23,24]. Notwithstanding its significance, automated segmentation remains challenging for several widely-used non-fluorescence, interference-based microscopy imaging modalities (e.g., contrast microscopy) [25,26].

Nonetheless, microscopy alone is not sufficient for microorganism identifications for several reasons: small cells that are usually present are difficult to identify; prokaryotes vary widely in size and some cells are close to the resolution limits of the optical microscope; when observing natural samples, such cells can easily be missed, especially if the sample contains a large amount of particulate matter or a large number of larger cells; and it is often difficult to differentiate living cells from dead cells or cells from inanimate materials present in natural samples [17]. Additionally, the major limitation related to microscopy is that none of them reveal the phylogenetic diversity of the microorganisms present in the study habitat [17]. Although, when microscopic analysis is associated with other tools, it becomes more promising. In addition, there are techniques of electron microscopy that are powerful instruments in the identification of microorganisms: transmission electron microscopy (TEM); scanning electron microscopy (SEM), confocal microscopy (CLSM), and atomic force microscopy (ATM) [27,28]. These techniques have great value mainly in the identification of microorganisms in biofilms [29]. Furthermore, microscopic techniques are generally associated with fluorescent dyes, which makes the visualization more specific and easier to perform. As an example, DAPI (49,6-diamido-2-phenylindole) is a broad use for this purpose, as is acridine orange dye. There is also increasing use of SYBR^®^ Green I, a dye that imparts bright fluorescence to all microorganisms, including viruses. These dyes bind to the DNA and fluoresce strongly when exposed to ultraviolet (UV) arraying (DAPI absorption, at 400 nm, acridine orange absorption maximum, 500 nm, SYBR Green I, maximum absorption, 497 nm), causing the microbial cells to be visible [30,31]. Depending on the sample, background coloring is occasionally a problem for fluorescent dyes, however, as these dyes are specific for nucleic acids they mostly do not react with the inert matter. Thus, they can be used for many soil samples, as well as from aquatic sources. In addition, staining with SYBR^®^ Green I provides excellent identification of aquatic virus populations. For diluted aquatic samples, cells can be stained after collection on a membrane surface by filtration [32,33].

The inverted optical microscope with laser tweezers can also be used. These consist of a precisely focused infrared laser equipped with a micromanipulation device. It is possible to capture a single cell because the laser generates a force that pushes a microbial cell (or other small object) and holds it in place. When the laser beam is moved, the captured cell escorts it. If the sample is in a capillary tube, a single cell can be captured optically and away from the contaminating organisms. The cell can then be isolated by the broken tube in a region between the cell and the contaminants and the cell can then be inoculated into a small tube containing sterile medium [34,35]. When associated with the use of staining techniques capable of identifying, in particular, organisms, the laser tweezers can be used to select organisms of interest from a mixture for purification and subsequent laboratory study [35].

Individual cells can also be identified by flow cytometry, a technique for counting and evaluating microscopic particles by suspending them in a fluid flow and passing them through an electronic detector. This method evaluates selected criteria including the size, shape, or fluorescent properties of individual cells as they pass through an index detector of many thousands of cells per second, and it can also classify the individual cells based on measurement criteria. The latter capability of the flow cytometer can be used to enrich a particular cell type from a mixture of various types [36].

## 3. Biochemical Analytical Methods to Detect Microorganisms

### 3.1. Traditional Biochemical Methods

In microbiology, traditional identification methods rely mainly on cultivation proceedings employing various media to enumerate, isolate, and identify specific microorganisms. For many years these methods were employed extensively and they continue to be used nowadays, especially in some laboratorial routines where a particular type of microorganism has to be identified rapidly (for example, in a medical diagnostic for the detection of a particular pathogen). Although being inexpensive and allowing both quantitative and qualitative information about the diversity of microorganisms present in a sample, however, these methods are laborious and time consuming (media preparation, dilution, plating, incubation, counting, isolation, and characterization), and results are only observed after several days, and frequently false positives are obtained especially when considering similar microbial species [37,38]. Another problem associated with culture-based methods is the fact that they cannot identify non-culturable cells.

Phenotypic identification methods usually incorporate reactions to different chemicals. One of the traditional methods most used is a simple visual detection of growth of the tested organism in the presence of a substrate by increased turbidity. Results are determined by comparing the microbial under analysis with a control test, and a Wickerham card is used to read the turbidity [39]. This type of reaction may be difficult to read and always involves a minimum of an overnight incubation. Immunoassays such as the enzyme-linked immunosorbent assay (ELISA) [40], although efficient, are expensive and are designed for only some bacterial species. A typical model is the analytical profile index (API) (bioMérieux, Craponne, France), where standard methods are integrated into miniaturized reaction couples, scored as “positive” and “negative” and finally matched to a scoring system on the basis of “best fit”, to create an analytical profile [41]. For many years researchers have used API 20E (analytical profile index 20E) testing apparatus, which consisted of a plastic piece with 20 cupules that contain pH-based substrates allowing the identification of almost 100 taxa [42,43,44]. Until 1992, this method was considered the “gold standard” especially in clinical microbiology. A significant advantage of this method was the availability of an extensive database, although it had a major disadvantage associated with it being time-consuming. Other automatic methods started to appear in this decade including improvements of the API 20E system (for a more detailed review see [39]), in an attempt to reduce the time needed for the procedure using recurring automations. The BBL Crystal (Becton Dickinson, NJ, USA) [45] is also a variation of the API system. An automated version of the API is the Vitek^®^ system (bioMérieux, Craponne, France), first developed in the 1970s, which eliminated the subjectivity of the reading of test reactions [46]. The Vitek^®^ system is useful for simultaneous bacterial identification and antimicrobial susceptibility testing (AST) profiles from isolated patient samples [47]. The system uses a totally automated broth microdilution technique that applies attenuation of light measured by an optical scanner for growth or no growth detection (it is crucial that the samples in the cards are pure isolates) [48]. The device regularly monitors growth over a period of 18–24 h for bacteria and 36 h for yeast. Although it is versatile, there are some microorganisms that lead to correct MIC reports or yield unreliably (e.g., pseudomonas) [47,49].A variation of the Vitek^®^ is the Biolog OmniLog System. It is a rapid, standardized, method for determining bacterial oxidation (tetrazolium redox dye) of different and simultaneous carbon sources (sugars, carboxylic acids, amino acids, and peptides, where 71 are carbon sources and 23 are chemical assays counting pH, salt tolerance, and chemical sensitivity tests). The results obtained are compared to a database (through an analysis of the obtained “yes–no” reactions) [50]. It is available for the phenotypic identification of bacteria and fungi (filamentous and yeasts) [51].

With the advancement of biochemistry knowledge and the appearance of more robust instrumentation, these methods started to be used less and more modern biochemical methods were developed, with numerous advantages over conventional culture-based methods, such as short analysis times and the ability to simultaneously determine many microorganisms, while retaining accuracy of the results [37,39].

### 3.2. Mass Spectrometry-Based Methods

Research in microorganism identification has evolved mainly by following the strategy of reducing the time required for the identification of a particular microbial in routine diagnostics. For this, the use of semiautomatic and automatic systems based on biochemical methods was a major breakthrough in this area. For a method to be considered successful nowadays, the attainment of results should take a maximum of 24 h, and in an emergency there is an urgent need for this time frame to be even smaller. Methods based on mass spectrometry (MS) have gained popularity as a microbial typing tool due to their speed, reduced costs, simplicity, and applicability for a wide range of microorganisms such as bacteria, archaea, and fungi [52,53]. With the advancements observed in MS methods, along with new data analysis, and processing and visualization tools, our understanding of biological systems has also increased, since we could analyze diverse sets of biomolecules, such as proteins, lipids, carbohydrates, and amino acids [53,54,55,56,57]. Several ionization and separation techniques can be coupled with MS, such as gas chromatography (GC) [52,58,59,60,61], matrix-assisted laser desorption ionization time-of-flight mode (MALDI-TOF) [62,63,64,65,66,67,68], electromigration techniques [37,69], and electrospray ionization (ESI) [70,71].

#### 3.2.1. Liquid Chromatography: High Performance Liquid Chromatography (HPLC)-Based Methods

The combination of liquid chromatography (LC) with MS (LC-MS), despite initial hesitations, revolutionized analytical determination of metabolome, consequently, allowing microorganism identification, by enabling the analysis of non-volatile or thermally labile high molecular compounds where gas chromatography and mass spectrometry (GC-MS) approaches were not suitable [72,73,74]. LC partings compatible with ESI are required and usually due to the polar and ionizable characteristics of most metabolites [75].

Comparing LC-MS with GC-MS, the temperatures necessary are lower and the sample volatility is not mandatory, simplifying sample preparation and lowering costs. LC-MS is mainly used in clinical applications for microorganism identification [76], but it is also useful in the detection of commercially available compounds of the in silico metabolome of *Bacillus subtilis* and *Escherichia coli*, and determining the complete metabolome coverage of *Saccharomyces cerevisiae* [75]. In LC-MS, samples are injected into the solvent stream using the injector and are divided within the column to which the stationary phase is chemically bound. Then, the eluent in the column passes across a flow cell in a spectrometer for non-destructive recognition of compounds with spectrometric structures (chromophore or fluorophore).

Progress in LC-MS technology was made by advances in mass analyzers and in the ionization technique, which led to new platforms: fast LC-MS, LC-MALDI-MS, LC-ESI-MS-MS, LC- Nuclear Magnetic Resonance (NMR)-MS, hydrophilic interaction liquid chromatography (HILIC)-MS, reverse phase LC-MS and ion mobility spectrometry [77,78,79,80,81,82]. In fact, HPLC is derived from LC, but the working pressures are meaningfully higher. Whereas, normal LC relies on the force of gravity to pass the mobile phase through the column, and in this methodology pressures are classically between 50 and 350 bars. The sample mixture is brought by the sampler into the column and the desired flow is provided by the pumps. The detector creates a signal proportional to the quantity of sample component in the column and the digital microprocessor controls the instrument providing data analysis. Presently, it can be used in monolithic columns [83,84,85,86] or at higher temperatures—high temperature liquid chromatography [87,88,89]. Some concerns which have to be considered when choosing HPLC for microbial detection are that the equipment is expensive and it demands complex maintenance procedures, and therefore some caution is advisable for an adequate interpretation of results. Denaturing high-performance liquid chromatography (DHPLC) is a new and promising approach, especially regarding bacterial identification and monitoring [90,91]. This technology is an automated system that allows separation of PCR products using ion-pair, reversed-phase, high-performance, liquid chromatography (IP RP HPLC).

#### 3.2.2. Gas Chromatography–Mass Spectrometry

GC coupled to MS has been extensively used in the identification of complex biological mixtures [92,93,94]. The GC system includes a gas supply, an injector, and a column inside an oven, coupled to the mass spectrometer. The data analysis can be executed using constant flow or pressure or by a flow program. While MS delivers individual mass spectra that can distinguish amongst chemically diverse metabolites, GC has a good separation efficiency. Additionally, GC-MS offers sensitivity, robustness, easiness of application, low cost, and abundant linear range, as well as commercial and public libraries accessibility [95,96,97]. This method is generally acknowledged for nonpolar molecule analysis (e.g., lipid components) [98], and is used for classification of microorganisms via assessment of their lipid elements. For instance, Curie-point pyrolysis-MS (Py–GC-MS) is applied for the distinction between Gram-positive and Gram-negative bacteria [99]. In addition, Ishida et al. [100] used thermally assisted hydrolysis and methylation-GC and MALDI-MS combined with on-probe sample pretreatment to directly analyze *E. coli* K-12 phospholipids in whole cells. In yeasts, this analysis has also been shown to be possible [101] for several species [102,103,104]. 

The main drawback of GC-MS is that it demands that the analytes are in a volatile form and as several metabolites are nonvolatile, time-consuming derivatization steps are required [105,106,107]. To optimize the technique’s performance, there are some technologies that can be conjugated with GC-MS, for example, GC-GC time-of-flight (TOF)-MS [108,109]. In this case, two different GC columns are conjugated, increasing the metabolite detection coverage, and the speed of scanning rate (TOF-MS) with extra sensitivity for improved detection. However, this method has high costs, so it is not yet routinely used. The connection of the flame ionization detector (FID)–GC-FID can also be applied for routine sample analysis, being rapid, very sensitive, and with an associated lower cost [110].

#### 3.2.3. Matrix-Assisted Laser Desorption/Ionization (MALDI)-Time-of-Flight (TOF)

MALDI-TOF MS is the latest next generation tool being used for the rapid identification and classification of microorganisms. It is based on the ionization of the microbial cells with short laser pulses and then accelerating the particles in a vacuum system using an electric field [56,66]. After the ionization, a molecular fingerprint in the form of a spectra profile is obtained, which is specific for each microorganism. This spectrum is then compared to an existing database, resulting in its identification by an automated program. Preparation of samples for MALDI-TOF MS involves crystallization with a large molar excess of matrix (usually a UV-absorbing organic acid) on target plates [62].

An alternative to MALDI-TOF which sometimes entails problems associated with the use of a chemical matrix (mixed with the sample) and the laser (used to effect desorption and ionization of the analyte), is a technique called electrospray ionization (ESI)-MS that analyzes samples in a liquid state and the ionization is carried out at atmospheric pressure, without recurring to the same lasers as in MALDI-TOF-MS. Due to this particular aspect, ESI-MS has a large spectra of applications regarding microbial identification [71,111,112,113].

### 3.3. Spectroscopic Methods

Fiber optics spectroscopy is a powerful multivariate and reproducible methodology, showing great potential in today’s research. It is used in systems biology, being a nondestructive, very simple, and to some extent, precise approach, allowing vast amounts of information to be obtained in one measurement [114,115]. Fiber optics spectroscopy processes vibrations and rotations of molecular functional groups, which are outcomes from the energy shifted when radiation interacts with a sample, and originates electronic excitation, vibrational change, and rotational change. The spectra will vary depending on the sample molecular groups, and therefore, they are linked to their chemical composition (proteins, lipids, carbohydrates, membranes, pharmaceuticals, human tissues, among others (reviewed in [116]). Fluids, cells or tissues can be explored to find metabolic fingerprints, and in theory, any sample can be virtually analyzed by spectroscopy. With respect to the identification of microorganisms, these methods are of great value and complementary to molecular biology, because they do not normally need the destruction of the sample. In the recent past due to some limitations found, some caution was advised by some authors when using these methods, suggesting a careful validation of each procedure before its use.

Numerous techniques for spectroscopic analysis are accessible. The subdivision is not always easy, varying from the type of radiative energy, the nature of the interaction or the material below analysis. 

#### 3.3.1. Infrared Spectroscopy (FTIR)

Recent advancements have been made especially in the application of new spectroscopic methods. One of these methods, with great developments in several areas of microbiology, is the Fourier transform infrared spectroscopy (FTIR). FTIR is versatile, fast, non-invasive, and it is easy to perform compared to other methodologies [114,117,118,119,120]. This analytical technique is a chemical and label-free procedure which gives a clear elucidation about the chemical composition and the physical state of the entire sample where several biomolecules can be analyzed. With the use of only a minimal amount of sample, it is possible to obtain, in a single measurement, detailed information about the main biomolecules such as lipids, proteins, carbohydrates, and nucleic acids [121]. Likewise, FTIR allows an economic biochemical characterization of complex biological systems, comprising the intact cells, tissues, and even whole-model organisms [122].

The use of this technique to evaluate microorganisms as biological systems results in a very complex spectrum with the overlapping absorption bands of the principal compounds. Therefore, a proper multivariate statistical analysis is of crucial importance in order to extract from the spectra only the relevant information of the biological process under study [122]. FTIR spectroscopy of biological systems provides a complex infrared absorption spectrum that should be preprocessed and then analyzed by applying resolution enhancement approaches. With respect to the operation mode of this technique, it comprises an IR energy source that emits a broad band of distinct wavelengths. After this, the radiation passes through an interferometer responsible for modulating the wavelength of IR. In the sample compartment, the resulting IR beam is absorbed in distinct and specific wavelengths by the organic or inorganic material. The last step comprises the measurement of the intensity of the IR beam by a detector producing an interferogram, that is, subsequently, analyzed by a computer using Fourier transforms, giving rise to an IR spectrum. Additionally, with the use of second derivatives it is possible to promote a clear separation of the absorption components, thus helping to understand their variations throughout the biological process under study. Subsequently, an adequate multivariate analysis should be implemented to validate the spectroscopic results, as well as to identify the main relevant bands of the process studied. Finally, the interpretation and analysis of the spectral data should be coupled with other standard methods to ensure the reliability of FTIR spectroscopy analysis [122]. Some of the principal advantages of this spectroscopic technique are related to: (I) the possibility of analyzing several compounds at the same time; (II) the facility of sample preparation, since it does not require cell lysis to release the biomolecules to be evaluated; (III) the association of an environmentally friendly role, as the toxic compounds are not implemented in this method; and (IV) the possibility of using this technique for real-time process monitoring and the accomplishment of high-throughput screenings [121]. Other regions of the infrared spectrum can be used in combination with spectroscopy to analyze microbial diversity. In particular, considering the infrared spectral regions, the wavelength region between 0.78 and 1000 µm can be divided into five subregions. However, the most relevant for spectroscopic purposes are the near-infrared, the mid-infrared (MIR), and the far-infrared radiation [123].

#### 3.3.2. Raman Spectroscopy–Vibrational Spectroscopy

Another spectroscopic method largely used is Raman spectroscopy, which has gained a wider acceptance as a mature analytical tool for the non-invasive and rapid characterization and identification of microbes during roughly the last 15 years. It discriminates itself from other systems by the manageability of use at a low cost, high speed, and an extensive report (chemical composition, the structure, and interactions of biomolecules in the microorganisms) [124,125]. In fact, this procedure uses vibrational, rotational, and other low-frequency modes in the system in order to produce a structural fingerprint by which molecules can be identified, providing complementary information to traditional spectroscopic methods, being many times, as will be described later, advantageous to combine several spectroscopic methods together. The structural fingerprint obtained is then used to identify microorganisms, as this method is capable of correctly distinguish between species and strains within a few hours. Although this high specificity is attributed to Raman spectroscopy, its sensitivity is rather poor. As in other spectroscopic characterizations, Raman spectroscopy depends on its interaction with the atoms and molecules, when light is incident on the matter. When atoms vibrate it will change the polarizability of functional groups, having nonpolar groups such as C-C and S-S intense Raman bands. Raman spectroscopy evaluates the inelastic scattering of radiation of monochromatic light, promoting a spectral shift, i.e., “Raman” shift, which scores from the interface of light with electron clouds surrounding molecular bonds [126,127]. Raman spectroscopy has been largely applied to microbial identification in recent years [126,127,128,129,130].

Both infrared and Raman spectroscopies are forms of vibrational spectroscopy and can provide “whole organism fingerprinting” as stated by [126]. Because vibrational spectroscopy discriminates microorganisms based on their biochemical composition, it is very useful for differentiating between minor differences among the same species.

#### 3.3.3. Nuclear Magnetic Resonance (NMR) Spectroscopy

NMR spectroscopy is an alternative and potent technique for microorganism identification. Strong magnetic fields and radio frequency pulses to the nuclei of the atoms are applied and in the case of atoms such as ^1^H or ^13^C, the magnetic field will cause a nuclear spin, absorbing the radio frequency energy (low-energy to high-energy spin states), and the emission of radiation is detected [131]. As compared with other methods, NMR can be performed in a non-invasive manner. Its sensitivity is reduced and it has a lower limit of detection (about 1–5 µM and a requirement for relatively large sample sizes of ~500 µL), although these issues are balanced by that fact that it is a quantitative method [132].

### 3.4. Electrokinetic Separation Methods

The term electrokinetics refers in science to the relative motion of a charged particle through a matrix. These methods make use of the differences in microbial composition to obtain different migration patterns, and in this way, without recurring to sample labelling, separate different microbial species.

Capillary electrophoresis (CE)–MS was developed and first published in 1989 by Joseph Loo. It combines the separation process of electrophoresis with MS detection [133]. In comparison with GC and LC, it includes better separation efficiencies, the use of very little sample volumes, speed, small reagent costs, and the possibility to separate cations, anions and uncharged molecules in a single run. This approach has been used to analyze the metabolome of numerous microorganisms, both for target and nontarget studies, having interesting outcomes in detection and quantification of several metabolite classes [134,135] (e.g., inorganic ions [136], organic acids [137], amino acids [138], nucleotides/nucleosides [139], vitamins [140], thiols [141], carbohydrates [142], and peptides [143]). CE has deficient sensitivity related to the small sample volumes, particularly when attached to MS, has a restricted quantity of accessible commercial libraries, and reduced retention time reproducibility.

Armstrong et al. [144] combined CE with capillary isoelectric focusing (CIEF) to separate and identify seven microbial species with a wide range of sizes and shapes. The uniqueness of this study was that it demonstrated that intact biological cells could be efficiently separated by employing techniques that are usually limited to macromolecules. Nowadays, another possibility used is the combination of CE with fluorescence, which can be used to observe the separation process, and in this way monitor the operational conditions and the microbial dynamics in terms of cell aggregation and focusing effects [145,146,147].

The main advantage of these types of techniques which focus on electrokinetics is the possibility to exploit several microbial parameters, such as size, shapes, and charges, which are very advantageous to their separation and identification.

Electrical field-flow fractionation (EIFFF) is another technique that uses the ability of microorganisms to migrate in an electric field. Its use for microbial identification was confirmed in 2000 [148]. It is based on the separation of sample components in a channel as a result of different layers (fractionation) of each group of components under the influence of various electrical fields. The EIFFF apparatus uses the two main walls of the channel to create a difference in the potential between the electrodes which leads to a separation between charges [149].

### 3.5. Microfluidic Chips

Since its appearance in the early 1990s, the microfluidics field of research has seen great and rapid developments [150]. It is a technique that combines separation and detection of sample constituents by controlling the movement of fluids within microfluidic chips, without the need for special sample preparation or reaction [151,152]. These platforms are small portable devices that combine microchannels (with dimensions from tens to hundreds of micrometers), pressure systems, and detection systems in the same piece. 

Several reviews have been published over recent years about the plenitude of microfluidics application [153,154,155,156,157], although, in light of this review, the development of microfluidics chips to detect microorganisms are the most relevant [152,158,159,160]. Detection of pathogenic bacteria and viruses using Chips is possible by recurring small sample volumes with great sensitivity, and it has huge applicability in food safety control, environmental monitoring, and clinical diagnosis. Recent approaches for microbial detection and identification are focusing on combinations of analytical standard techniques within microfluidics chips, without the need for labeling procedures. In particular, there are several reports of combinations of microfluidics devices with PCR [161,162], MS approaches [163,164,165], spectrometry [166], electrochemistry [154,167], among others.

FISH (fluorescence in situ hybridization) technique involves the use of phylogenetic dyes which are fluorescent oligonucleotides whose base sequences are complementary to the ribosomal RNA sequences (16S or 23S RNA in prokaryotes, or 18S or 28S RNAs in eukaryotes). These phylogenetic dyes have the ability to penetrate into cells without promoting their lysis, and within the cell they are able to form hybrids with the microbial ribosomal RNA. Given that the ribosomes are distributed throughout the cell in prokaryotic organisms, the whole cell becomes fluorescent [168]. These dyes are generally specific, reacting with only one species or a few related microbial species, as well they produce more generally and react with, in some cases, all cells of a given phylogenetic group. It is important to highlight that the use of this technique allows the identification and search of an organism, or domain of interest, that is present in a natural sample [169]. FISH technology can also use various phylogenetic probes. Thus, one can use a set of probes where each is designed to react with a particular organism or group of organisms, where each contains its own fluorescent dye. With FISH it is possible to determine the phylogenetic amplitude of a single habitat in a single experiment, and by associating FISH with CLSM, it is possible to study microbial populations in greater detail and use it in biofilm study [30]. Additionally, FISH technique can be used to measure the gene expression of the organisms present in a natural sample. In this case, since the target corresponds to mRNA (less abundant than the rRNA present in the ribosomes of a cell) standard FISH techniques cannot be applied, and instead the target mRNA or fluorescence signal must be amplified [170].

## 4. Molecular Methods Used to Detect Bacteria

The advent of the “molecular biology age” has provided a plethora of tools and techniques for the detection, identification, characterization, and typing of bacteria for a range of clinical and research purposes [171]. Previously, the identification and characterization of bacterial species was largely done by phenotypic and biochemical methods, which relied on preliminary isolation and culture. While these methods continue to hold place in certain settings, molecular-based techniques have provided unprecedented insights into bacterial identification and typing. To name a few examples, genotypic methods have enabled the identification of a large diversity of previously unknown taxa, the characterization of uncultivable bacteria, and facilitated metagenomics studies on large and diverse bacterial communities [172]. Both clinical and research setting have provided in depth insights into bacterial virulence, pathogenesis, antibiotic resistance, and epidemiological typing, as well as identification of novel, emerging, and re-emerging species [173]. In addition, the widespread use and availability of molecular tools for bacterial genotyping has resulted in high throughput analysis, more sensitive and discriminatory results, and rapid turn-around-times, which are only likely to get better with automated tools and data analysis pipelines. Most molecular methods for bacterial identification are based on some variation of DNA analysis, either amplification or sequencing based. These methods range from relatively simple DNA amplification-based approaches (PCR, real-time PCR, RAPD-PCR) towards more complex methods based on restriction fragment analysis, targeted gene and whole-genome sequencing, and mass spectrometry. In addition to this, approaches based on unique protein signatures such as matrix-assisted laser desorption/ionization time-of-flight mass spectrometry (MALDI-TOF-MS) and similar variations have also been explored [174]. While the advantages and limitations of these approaches vary, the choice of the technology employed depends on several factors including sample type (clinical or research, single-species or mixed-species), depth and accuracy of results generated, resources and cost factors, as well as the turn-around-times expected. Given that the present “molecular biology revolution” is resulting in a larger number of laboratories, including small-scale and resource-limited setups, having access to genomic approaches, it is imperative to understand the fundamental principles of these techniques, their applications, and their limitations.

### 4.1. 16S rRNA PCR-Sequencing

The rapid amplification of nucleic acid targets from relatively lower starting material, makes PCR one of the most sensitive techniques available for detection of bacterial targets. PCR-based identification of bacterial DNA through amplification and sequencing of the 16S rRNA gene has become a standard molecular method, both in the laboratory as well as in clinical settings. The 16S rRNA gene is highly specific to each bacterial species and this makes it an ideal target for identification. The standard method involves PCR amplification of the 16S rRNA gene, followed by sequencing and comparison to known databases for identification. PCR-based methods are not only faster than conventional culture-based methods but are also helpful in identification of bacteria that are difficult to grow in laboratory conditions. In one study, universal primers for the 16S rRNA gene were designed to identify bacteria in the root canals of patients with necrotic pulp tissue [175]. The primers included ten putative bacterial pathogens commonly found in root canals with necrotic pulp. After DNA extraction from the necrotic pulp, a PCR was run using universal primers, as well as species specific primers, and the products were analyzed using gel electrophoresis. Twenty-two of the 24 specimens tested positive with the universal bacterial primers. As expected, certain bacterial species such as *Fusobacterium* spp., *Peptostreptococcus* spp. and *Streptococcus* spp. were commonly identified. Of these, PCR analysis revealed two samples that showed a product with the universal primers, but not with any of the 10 species-specific primer sets tested. Sequencing of these PCR products revealed the presence of a close relative of the *Olsenella* genus, previously not associated with such infections. Though the 16S rRNA gene has emerged as a popular target for PCR-based identification, in cases where the 16S rRNA gene is identical in two closely related species, other conserved genes, such as rpoB, tuf, gyrA, gyrB, and heat shock proteins are used as targets [176,177,178]. In research laboratories, PCR-based identification is a straightforward procedure with reliable results. However, when applied to clinical settings, various factors come into play that can influence PCR results. Clinical samples often have very few bacteria to begin with, they also require various preprocessing steps before the PCR is carried out, to remove PCR inhibitors and enable extraction of maximum bacteria from the sample without contamination [179,180]. Despite these concerns, PCR-based identification has been successfully and widely employed to detect and identify bacteria in clinical samples [181,182,183].

### 4.2. Real-Time PCR

Real-time PCR (RT-PCR) provides many advantages over conventional PCR, such as higher sensitivity and accuracy and the ability to monitor DNA amplification in real-time through fluorescence intensity, thereby negating the need for any post-PCR detection techniques. RT-PCR can also be quantitative or semi-quantitative, using the Cq value (cycle number at which fluorescence intensity rises above the detectable level) to quantify the amount of DNA. As with conventional PCR, RT-PCR also has wide applications within research laboratories as well as in the clinic. It is also applied to numerous kinds of samples, right from identification of bacteria in milk, which are otherwise non-culturable [184], to the identification of bacteria within soil ecosystems [185]. Using universal primers that target conserved regions of 16S rRNA gene, an assay has been developed that can detect *Acinetobacter baumannii*, *Escherichia coli*, *Klebsiella pneumoniae*, and *Pseudomonas aeruginosa* [186]. The primers were designed based on the alignment sequence of 962,279 bacterial 16S rRNA gene sequences, which revealed two regions that were highly conserved in more than 90% of all rRNA gene sequences. The primers were able to successfully detect less than 100 genomic DNA copies. This real-time based 16S rRNA PCR has also been used to identify or quantify bacterial loads in clinical infections such as chronic wound tissue [187] and gastrointestinal mucosal biopsies [188], and has also been applied in forensic investigations of saliva specimens [189]. In addition, high resolution melting (HRM) is a rapid, reliable, accurate, and cost-effective emerging tool for genotyping bacteria, such as from the *Lactobacillus casei* group and both Gram-positive and Gram-negative bacterial pathogens [190,191].

### 4.3. Random Amplification of Polymorphic DNA–RAPD-PCR

Unlike previously described PCR-based methods, random amplification of polymorphic DNA (RAPD)-PCR employs short primers (8–12 nucleotides long) with arbitrary sequences that bind nonspecifically to template bacterial DNA. This results in amplification of random, repetitive regions of template DNA, thereby providing a unique profile for bacterial identification [192]. RAPD-PCR reactions can start from isolated DNA or crude bacterial lysates, which are then subject to amplification in the presence of a RAPD primer (or set of primers) and low levels of magnesium (to enhance non-specific annealing) [193]. Amplified products are then subjected to standard agarose gel electrophoresis to generate unique RAPD fingerprints. RAPD-PCR requires no prior knowledge of the target genome sequence, as the primers are designed to bind randomly to the template DNA. This means it can be used to identify and type a diverse range of bacterial species that have either not been identified or for which no prior sequence data is available. Furthermore, it can be performed from whole bacteria directly, without the need for DNA isolation, and can be applied on Gram-positive and Gram-negative species [193]. In India, 20 alkaline protease producing bacterial strains isolated from soil samples from various geographic regions were subjected to RAPD screening with a set of random primers [194]. Analysis of the amplification pattern enabled the classification of isolates into distinct groups based on alkaline protease production. This underscores that RAPD typing using universal random primers are a viable alternative to gene specific molecular marker identification, especially when analyzing a large number of samples of diverse species and without any prior genetic information. Lastly, RAPD-PCR has also been used as a tool to identify the genetic variability of microorganisms [195,196,197].

### 4.4. Restriction Fragment Length Polymorphism–RFLP

Restriction fragment length polymorphism (RFLP) is a method for identifying bacterial strains using unique fingerprints which relies on the presence of variations (polymorphisms) in homologous DNA sequences. This PCR-based method employs restriction enzymes, which can recognize and cut amplified DNA (PCR product) into DNA fragments of different lengths. As in RAPD, these different fragments are separated by agarose gel electrophoresis to generate a unique pattern of bands for each bacterial strain. If two strains are closely related, their banding patterns will be identical or very similar. On the other hand, differences in banding patterns indicate bacterial strain diversity. As evident, this technique is highly relevant in investigating the molecular epidemiology of infectious outbreaks, where it is important to establish whether multiple cases or patients belong to the same outbreak, to track the source of the outbreak, and to determine single or multiple bacterial strains involved in the outbreak. In a suspected nosocomial outbreak of methicillin-resistant *Staphylococcus aureus* (MRSA), PCR-RFLP identified three novel MRSA isolates based on new RFLP patterns. Further, in a smaller group of patients, the highly discriminatory nature of PCR-RFLP analysis was able to correctly identify differences in a cluster of MRSA strains, thereby ruling out the possibility of an outbreak [198].

### 4.5. Amplified Fragment Length Polymorphism–AFLP

Amplified fragment length polymorphism (AFLP) is similar to RFLP, in that it employs restriction enzymes (usually a pair) to fragment genomic DNA, but then amplifies a subset of restriction fragments using ligated adaptors. This amplification is achieved by using primers that are complementary to the adaptor sequences but also have certain unique nucleotides. Therefore, only a small number of restriction fragments are selectively amplified. AFLP fingerprints are then analyzed using gel electrophoresis, yielding a set of distinct DNA fragments from a single bacterial genomic DNA. As evident, AFLP offers high specificity and discriminatory potential in the absence of any prior genome sequence knowledge. The advantages of AFLP as a DNA fingerprinting tool were leveraged in an outbreak investigation of *Pseudomonas aeruginosa* in an intensive care unit [199]. During a period of one year, 23 *P. aeruginosa* strains from infected ICU patients were isolated and characterized by AFLP. Following restriction digestion, AFLP PCR was performed using fluorescently labeled PCR primers. Fluorescent amplified PCR fragments were separated by gel electrophoresis and analyzed. Based on the AFLP results, an outbreak cluster was identified with more than 90% similarity. Notably, this outbreak strain was also isolated from the wash basin, water tap, and connection pieces from suction tubes, pointing to the possible source of the outbreak [199]. Complete elimination of the outbreak was achieved after sterilization of the ICU equipment.

### 4.6. Pulsed-Field Gel Electrophoresis–PFGE

Pulsed-field gel electrophoresis (PFGE) is a method of separating large fragments of DNA and is particularly useful for characterizing and typing bacteria for epidemiological studies. In PFGE, pure bacterial strains in agarose plugs are treated with enzymes and detergents (proteases and SDS) that release chromosomal DNA. The agarose plugs are then incubated with restriction enzymes, which cut at specific sites to generate a limited number of DNA fragments. The plugs are then subjected to electric current and alternate rotations in a magnetic field (which enhances the movement of large DNA fragments), leading to the size separation of DNA fragments and emergence of a banding pattern [200]. In an outbreak investigation of cholera over seven years, fifty isolates of *Vibrio cholerae* were subject to molecular typing by PFGE [201]. Analysis revealed that over the years, the outbreak involved 15 different pulsotypes of *V. cholerae*, four pulsotypes matched published pulsotypes and there were 11 new types. Notably, PFGE typing revealed the chronological emergence of new types, which subsequently replaced the earlier pulsotype.

### 4.7. Ribotyping

Ribotyping is a method for bacterial identification and characterization that, unlike certain previously described molecular typing methods, employs rRNA based phylogenetic analysis. Given that that rRNA genes (such as 16S rRNA) are highly conserved within a bacterial species, identifying 16S rRNA gene polymorphisms is a reflection of the evolutionary lineage of the bacterial species, and can shed light on bacterial classification, taxonomy, epidemiological investigation, and population biology [202]. Ribotyping typically involves a multi-step process starting with restriction enzymes that target the genomic sequence of interest, followed by southern blot transfer and hybridization with probes, and analysis of ribotype RFLP bands. However, with advances in molecular tools and knowledge of genomic sequences, several modifications to this technique have been published [202]. It is important to note that for the purpose of primer and probe design, ribotyping requires some prior knowledge of the genome sequence under study. In one study, PCR-ribotyping was employed to characterize 99 strains of *Clostridium difficile* isolated from patients with nosocomial diarrhea. Following DNA extraction and PCR amplification of select regions of the 16S rRNA and 23S rRNA genes, amplified products were fractionated by electrophoresis [203]. The banding pattern revealed 41 different PCR-ribotypes with high reproducibility and discriminatory power. In a modification of this method, PCR-ribotyping was directly employed on stool samples for detection and typing of *C. difficile* strains [204]. Primer modifications targeting both, the 16S-23S rRNA intergenic spacer region and 16S and 23S genes itself, resulted in increased specificity for direct typing. With these new primers, PCR-ribotype could be detected directly from stool samples in 86 out of 99 cases, with a high degree of concordance with PCR-ribotyping done from isolated colonies.

### 4.8. Whole-Genome Sequencing–WGS

Whole-genome sequencing (WGS) has recently become a highly accessible and affordable tool for bacterial genotyping. Analysis of the entire bacterial genome not only provides unprecedented insights into bacterial typing and evolutionary lineages but has also revolutionized our approach to understanding antimicrobial resistance and outbreak investigations. Advances in WGS technologies and analysis pipelines have rapidly increased output and analysis speed, while reducing overall costs [205]. In spite of reservations from clinicians related to experimental protocols and cost factors, WGS-based approaches are being evaluated for the pathogen identification and antimicrobial resistance typing. In one study, WGS was used to investigate a fatal outbreak of vancomycin resistant *Enterococcus faecium* (VRE) involving three patients in an ICU [206]. Using an Illumina Miseq benchtop sequencer, WGS established that isolates from patient two and three differed from that of patient one only by a single, non-synonymous polymorphism, each pointing to ICU transmission. In addition, the distinct SNPs in isolates from patient two and three also indicated two separate direct transmission events from patient one, rather than linear transmission from patient one to patient two to patient three. As expected, the isolates were shown to carry genes (vanA) conferring resistance to vancomycin. Therefore, the in-depth analysis offered by WGS was not only able to establish antibiotic resistance but could also infer transmission dynamics and evolutionary lineage of the outbreak strains.

### 4.9. MALDI-TOF-MS in Bacteria

In addition to advancements in genomics, proteomics-based approaches for bacterial identification and characterization have emerged. These methods are primarily based on mass spectrometry, which enables rapid and high-throughput analysis of biomolecular signatures produced by a bacterial strain [174]. In MALDI-TOF MS, the spectra patterns produced from bacterial cells contain characteristic information to identify and characterize bacterial species. For this, the bacterial sample to be analyzed is mixed with organic matrices and ionized by a laser beam. As the resulting ions move towards the mass analyzer, the mass:charge ratio is obtained which creates a spectra pattern. This pattern is then compared with a known library of fingerprints. In a sophisticated work, Edwards-Jones and colleagues [207] developed a MALDI-TOF-MS based method to discriminate between methicillin-sensitive and methicillin-resistant *S. aureus*. Based on the distinct spectral patterns obtained, MSSA and MRSA could be rapidly differentiated, and this was determined to be highly reproducible. This not only underscores the relevance of mass spectrometry-based approaches for bacterial identification and typing, but also indicates that it could assist with clinical decisions such as the initiation of appropriate antibiotics for the treatment of *S. aureus* infections.

## 5. Molecular Methods Used to Detect Yeasts

Rapid and precise identification of pathogens from clinical specimens leads to appropriate therapeutic plans [208], but the growing diversity of infectious species and strains makes the identification of clinical yeasts increasingly difficult [209]. Still, novel identified fungal species can differ in virulence and drug resistance. Culture-based identification methods have been the gold standard for the diagnosis of fungal infection [210], but these classical phenotypic and biochemical assays are time consuming and are not suitable to accurately distinguish all the species belonging to a specific cryptic complex [211]. Therefore, several molecular biology approaches have gained great potential, as they can be applied to detect the pathogen directly without prior cultivation to identify species and subspecies [212] and they go further than biotype or serotype [213].

In fact, these methods range from simple PCR methods to more sophisticated quantitative RT-PCR and/or matrix-assisted laser desorption time-of-flight mass spectrophotometry (MALDI-TOF MS) [211]. Molecular methods are based on the detection of the nucleic acid sequence of a gene specific to an organism, and therefore they do not detect viable organisms, only indicate their presence [214]. The principle of the probe-based identification is to obtain a double-strand hybrid as a result of binding the single stranded DNA or RNA of the organism to a complementary sequence. For molecular detection of fungal pathogens, PCR is the most preferred method [215,216] and is regarded as a standard platform in many clinical laboratories, even in developing countries, due to its affordability and reproducibility [217,218]. RT-PCR assays with a short turnaround time can provide desirable alternatives for the rapid detection of microbes [210,219], and they are able to quantify the amount of amplified DNA in real time. Hence, conventional PCR methods have been replaced by RT-PCR techniques in medical laboratories [216]. DNA fingerprinting methods have evolved as major tools for identification in fungal epidemiology. However, it must be emphasized that no single method has emerged as the method of choice, and some methods perform better than others at different levels of resolution [220]. DNA polymorphisms of medically relevant fungi can be detected by the analysis of RFLP, and southern hybridization with appropriate DNA probes, DNA RAPD, other PCR-based methods, electrophoretic karyotyping by pulsed field gel electrophoresis (PFGE), and sequencing-based methods [212,220]. The number of laboratories now using the relevant molecular testing is rapidly increasing, resulting in an obvious need for standardization. The application of the appropriate technique depends on factors such as financial budget, experienced personnel, and equipment but an important issue is still the lack of sufficient species-specific primers [214].

### 5.1. PCR

Aforementioned PCR-based detection of fungal DNA sequences can be sensitive, rapid, specific [212,221,222] and it permits both intraspecies differentiation and species identification of yeast isolates [223]. A relevant step for PCR performance is the DNA extraction, which should be universal, which originates pure and high-quality DNA [224]. Because fungal cell walls are strong and difficult to disrupt, DNA isolation requires effort to overcome this barrier. Hence, glass beads are used for mechanical disruption, sonication, and phenol-chloroform in order to promote enzymatic digestion in the lysis phase [216,225]. As previously indicated, PCR tests, as well as detection of specimen type (whole blood, serum, and plasma), should be standardized. The choice of primers is another important factor that could alter the diagnostic performance of PCR tests. On the other hand, multiplex PCR can detect a wide variety of fungi at once in the same sample [216]. The 18S, 5.8S, and 28S nuclear rRNA genes are coding regions with slow evolution and are relatively conserved among fungi. Because of these properties they provide establishment of phylogenetic relationships [222,226]. More rapidly evolved regions are internal transcribed spacer 1 and 2 (ITS1 and ITS2, respectively) and thus they may vary among various species within a genus. ITS region DNA sequences with sufficient polymorphism may be amplified in PCR and serve for fungal identification [222]. 

The abovementioned conserved sequence of 18S-rRNA was used for primer design with the goal of detecting 25 fungal species, including *Candida* spp., *Hansenula* spp., *Saccharomyces cerevisiae*, *Cryptococcus neoformans*, *Trichosporon beigelii*, *Malassezia furfur*, *Pneumocystis carinii*, *Aspergillus* spp., and *Penicillium* spp. A 687-bp product was amplified successfully by PCR from all 78 strains and specificity was subsequently confirmed by Southern analysis [221]. Stepwise “YEAST PANEL multiplex PCR assays” targeting 21 yeast species of *Candida* spp., *Trichosporon* spp., *Rhodotorula* spp., *Cryptococcus* spp., and *Geotrichum* spp. was designed as a faster and accurate diagnostic strategy. Primers were designed to not cross-react with the other species, AND compatibility of amplicon sizes of one target species with the rest of target species in the same multiplex PCR was required as well as melting temperature compatibility of primers within the same multiplex PCR. Another two criteria for primer selection were the location of primers in the most stable segment of the target loci, and in order to prevent cross-reactivity with nontarget species, the gaps, and mismatches (positioned in the 3′ end of primers). In this work, the results obtained from the YEAST PANEL multiplex PCR assay (with 804 clinical species) were 100% consistent with those of MALDI-TOF MS [218]. A multiplex PCR strategy, which allowed the identification of eight clinically relevant yeasts of the *Candida* genus, namely *Candida albicans*, *Candida glabrata*, *Candida parapsilosis*, *Candida tropicalis*, *Candida krusei*, *Candida guilliermondii*, *Candida lusitaniae*, and *Candida dubliniensis*, was focused on the amplification of fragments from ITS1 and ITS2 regions by the combination of two yeast-specific and eight species-specific primers (EMBL/GenBank database) in a single PCR reaction [227]. Another one-step, multiplex PCR to detect and identify *Candida* spp. in clinical settings was developed with primers targeting Hyphal Wall Protein I gene for the *C. albicans*, *C. dubliniensis*, *Candida africana*, Intergenic Spacer for the *C. glabrata*, *Candida nivariensis*, *Candida bracarensis*, and Intein and ITS rDNA for the *C. parapsilosis*, *Candida orthopsilosis*, and *Candida metapsilosis*. No cross-reaction with closely- and distantly-related yeast species, *Aspergillus* spp., and human DNA was observed, resulting in 100% specificity [218].

### 5.2. Quantitative Real Time-PCR

RT-PCR assay is an important tool for rapid detection of pathogens, and it offers superior accuracy and specificity over traditional methods. In a study, real-time amplification of two genes, melting-point analysis and two-dimensional plotting of T(m) data were used as a broad-range method for the identification of clinical isolates of *Candida* spp. and *Cryptococcus* spp. Two primer sets (18S-1F/5.8S-1R and 18S-2F/5.8S-1R) for amplifying ITS-1, one primer set (5.8S-1F/28S-1R) for amplifying ITS-2, and one primer set (28S-2F/28S-2R) for amplifying a variable segment of the 28S ribosomal gene were designed [228]. Conserved sequences DNA in *Candida* spp., however different from *Aspergillus* spp. and *Penicillium* spp., were identified. [210]. Further, species-specific real-time PCR primer sets covering *C. albicans*, *C. glabrata*, *C. tropicalis*, *and C. dubliniensis* were selected and they showed high sensitivity and specificity. They could be potentially assembled into a single PCR array for the rapid detection of *Candida* spp. in various clinical settings [210]. The same regions of the rDNA gene complex, the highly polymorphic ITS1 and ITS2, were amplified by another group using primers targeting conserved regions of the 18S, 5.8S, and 28S genes to identify fungal pathogens [228]. In another study, real-time PCR assay demonstrated to rapidly detect, identify, and quantify *Candida* spp. from blood culture samples [229]. The assay was performed with primers and probes specific for the 18S rRNA of *Candida* spp. A total of 50 strains, of *C. parapsilosis*, *C. glabrata*, *C. albicans*, and *C. tropicalis* were distinguished and the results were entirely in accordance with the sequencing and conventional methods [229]. In another approach, the inspection of the viability of a high-resolution melting curve analysis (HRMA) of regions ITS1 and ITS2 for the distinction of *C. albicans*, *C. glabrata*, *C. parapsilosis*, *C. krusei*, *C. tropicalis*, *C. guilliermondii*, *C. dubliniensis*, and *Candida lusitaniae* was considered. HRMA was verified in order to categorize *C. albicans* strains into four genotypes (A, B, C, and D) using a primer set that limits the transposable intron region of the 25S of rDNA. RT-PCR and HRMA produced clear melting curve shapes, according to sequencing and gel electrophoresis study, very reproducible, and typical of each *Candida* spp. and *C. albicans* genotypes [230]. Furthermore, Asadzadeh et al. [231] developed a duplex RT-PCR assay for rapid detection and differentiation between *C. albicans* and *C. dubliniensis* by using two species-specific primer pairs and SYBR^®^ Green dye to differentiate *C. albicans* and *C. dubliniensis* isolates via melting curve analysis of RT-PCR amplicons. The amplification products were also analyzed by agarose gel electrophoresis to confirm RT-PCR results. Melting temperature (Tm) for reference strains of *C. albicans* and *C. dubliniensis* were 86.55 °C and 82.75 °C, respectively [231]. Similarly, quantitative PCR assays to determine the relative *Paracoccidioides brasiliensis* load in lungs from infected mice were also developed. SYBR^®^ Green- and TaqMan-based assays using primers and probe for the 43-kDa glycoprotein (gp43) gene were able to detect as little as 270 gene copies (about 2 fg of DNA) per reaction. Although qPCR assays cannot distinguish between living and dead yeasts, it found a highly positive linear correlation between CFU and qPCR [219]. PCR and RT-PCR assays were also used to 100% identify 44 *C. auris* and related species, such as *Candida duobushaemulonii*, *C. haemulonii*, and *C. lusitaniae* with strong results [232]. Comparable scores were acquired when real-time PCR was applied as an amplicon with a Tm [232]. In another report, the RT-PCR also quickly revealed *C. auris* DNA from 1% and 12% of swab and sponge samples with culture-negative results, showing the presence of dead or culture-impaired *C. auris* [233].

### 5.3. DNA Fingerprinting Methods

#### 5.3.1. Pulsed Field Gel Electrophoresis (PFGE)

Electrophoretic karyotyping methods, which are based on differences in the genetic structure of an isolate, reveal sufficient variation for strain delineation [234]. Pulsed field gel electrophoresis (PFGE) enables separation of fungal chromosomal DNAs according to their size (up to several megabases) in agarose gels, and it is a worthwhile tool for fungal karyotyping [234,235]. Its application allows for species or even strain specific profiles to be obtained. For example, the chromosomal DNAs of eight *Candida* spp. were analyzed by PFGE under various conditions. The matching bands in the gels were appointed by DNA probe which hybridized to DNA of all the species (rDNA, TUB2, PEP4) [235].

#### 5.3.2. Restriction Fragment Length Polymorphisms (RFLP)

Delineating strains of *C. albicans* based on variations in DNA structure can be performed by RFLP. Concisely, DNA extracted from isolates is split into fragments by specific DNA restriction enzymes, and the fragments are divided based on molecular size by gel electrophoresis. To spot alterations or matches in the fragments a staining of the gel with ethidium bromide with visualization under UV light or DNA hybridization with a specific DNA probe is done [234]. Size and restriction scrutiny of PCR-amplified ITS2 region DNA is a fast and consistent routine to identify clinical yeasts. Presently, there is a validated database with over 400 clinical isolates (ITS2 length and sequence polymorphisms) for 34 yeast different species [222]. In another study, *Candida* spp. isolates (80 clinical isolates and three standard strains) from cancer patients were identified using other PCR-restriction enzymes, MspI and BlnI [236]. Alternatively, ITS-rDNA region (ITS1-5.8SrDNA-ITS2) was amplified by PCR using universal primers and the products were digested with MspI for identification of 360 clinical yeast strains from nail infection. Specifically, for the *C. parapsilosis* complex, the *SADH* gene was amplified, with a digestion done using the Nla III restriction enzyme. *C. albicans*, *C. parapsilosis*, *C. tropicalis*, *C. kefyr*, *C. krusei*, *C. orthopsilosis*, *C. glabrata*, *C. guilliermondii*, *C. rugosa*, and *C. lusitaniae* were identified [208]. The same method (RFLP based on Msp I and Bln I restrictive enzyme cuts PCR products after the amplification of ITS1 and ITS2 regions of rDNA genotypically) was used and results were compared with phenotypic species assessment using an automated Vitek^®^ 2 system. A great difference was found between these two methods. It may be argued that Msp I and Bln I restriction enzyme fragments can be used in the identification of medically important *Candida* spp. Further studies are needed to develop this kind of restriction profile to be used in the identification of candidal strains [237]. Restriction enzyme analysis of *C. albicans* and non-*Candida albicans Candida* spp. previously identified by conventional methods was done to evaluate the utility of restriction enzyme analysis for more rapid and reliable identification of 146 *Candida* spp. strains (MwoI for the totally of the species, and BslI for *C. parapsilosis* and *C. tropicalis* strains). The restriction digestion with MwoI was able to distinguish between five different species (*C. albicans*, *C. krusei*, *C. guilliermondii*, *C. kefyr*, and *C. glabrata*), while BslI digestion could distinguish between *C. tropicalis* and *C. parapsilosis* [238]. Another study summarized that phenotypic and molecular methods (PCR-RFLP) resulted in the identification of 65.2% and 96.6% of 204 *Candida* spp. isolates, respectively [239].

#### 5.3.3. Fragment Length Polymorphisms (RFLP)

Mitochondrial DNA (mtDNA) can also be useful to distinguish closely related strains in hospital acquired infection outbreaks since, as compared to nuclear DNA, its higher mutational load and evolutionary rate readily reveals microvariants [240]. Restriction endonuclease analysis of mtDNAs from 19 isolates representing seven *Candida* spp. and *Lodderomyces elongisporus* showed altered cleavage outlines that emerged to be specific for the species. Rare shared restriction fragments were clear and there was no correspondence among the base compositions of nuclear and mitochondrial DNAs. *C. parapsilosis* and *L. elongisporus* had similar mtDNA molecular sizes (30.2 and 28.8 kilobase pairs), however, the restriction endonuclease patterns of these organisms were distinct [241]. In another study, three intergenic regions located among the genes tRNAGly/COX1, NAD3/COB and ssurRNA/NAD4L, named IG1, IG2, and IG3, respectively, which showed a high number of neutral substitutions, were amplified and sequenced from 18 clinical isolates of the *C. albicans* strains, and phylogenies revealed three groups. Unbiased evolution, great variability, easy PCR isolation, and full length sequencing regions can lead to a novel outlook in molecular findings of *C. albicans* isolates, supplementing multilocus sequence typing techniques (MLST, [240]).

#### 5.3.4. Random Amplified Polymorphic DNA (RAPD)

RAPD markers are DNA fragments from PCR amplification of random segments of genomic DNA with single primer of an indiscriminate nucleotide sequence. RAPD or restriction enzyme analysis (REA) are valuable to establish the source of an outbreak, nonetheless, further reproducible and discriminatory procedures may be a requisite (e.g., Southern hybridization, PFGE). Multiple *Candida* strains from nosocomial infections have been identified [242]. By using OPE-18, OPE-04, and OPA-18, RAPD enabled a direct association of the most frequent *Candida* spp.(characteristic molecular fingerprint). In addition, the differentiation between *C. albicans* and *C. dubliniensis* and its strains were correctly performed by a PCR established on multiple secreted aspartic proteinase (SAP) and dipeptidyl aminopeptidase (DAP2) genes [243]. Moreover, genetic profiles of 39 clinical isolates of *C. albicans* were assessed by means of RAPD and microsatellite, with two different primers for each system. The identification of yeasts was set by nested-PCR which involved two amplification stages. RAPD afforded diverse profiles for both primers M2 and P4. Using CDC3 and HIS3 markers, microsatellite endorsed the observation of six and seven unlike alleles, respectively [244].

#### 5.3.5. Amplified Fragment Length Polymorphism (AFLP)

In this methodology, the genomic DNA is digested with two restriction enzymes (e.g., EcoRI and MseI) and double-stranded oligonucleotide adapters are linked to the fragments, which work as targets for the primers (labeled with a fluorescent dye ) during PCR amplification, separated, and scrutinized using a software [245]. In a collection of 395 clinical isolates catalogued as *C. parapsilosis*, 20 *C. metapsilosis* strains were identified by AFLP (polymorphic bands) [246]. About 104 *C. auris* isolates from India, South Africa, and Brazil were analyzed using MLST, AFLP fingerprinting, and MALDI-TOF MS (EcoRI and MseI restriction enzymes and complementary adaptors) [247]. Both MLST and AFLP typing clearly defined two major clusters covering isolates from India and Brazil [247]. All previously described molecular techniques can be applied for detection of new fungal species as well as for routine laboratory identification. For example, *Candida milleri* and *Candida humilis* are the most characteristic yeasts found in type I sourdough ecosystems. Genetic characterization, assimilation test of carbohydrates, and metabolome assessment by FTIR analysis exposed a high degree of intraspecific polymorphism and 12 distinctive genotypes were categorized [248]. Several methods were shown to be useful to determine isogenicity among *C. albicans* isolates obtained from the same patient: PFGE separation of chromosomes; RFLP of chromosomal DNA; and finally, Southern blot analysis [249]. *C. auris* isolates, identified by ITS rDNA sequencing demonstrated to be extremely resistant to fluconazole and resistant to voriconazole, stressing the importance of precisely identifying *C. auris* to avoid therapeutic failures [250].

## 6. Conclusions

The tools for the determining the identity of a microbial sample have been emerging in the last decades. Although having limitations, culture and microscopy are still two of the most utilized techniques. PCR and other genetic approaches are particularly important for nonculturable microorganisms and MS has been shown to be useful, quick, and easy for the identification of microbial samples and detection of microbial threats. However, it is reserved for pure isolates and cannot be used for complex samples, since they may promote interference in the background. This may be simplified through the use of chromatography-based methods (e.g., HPLC, LC-MS).

In the future, development of the detection limits for microorganisms will continue to be a key assignment in clinical microbiology. The combination of these (and possibly others) methodologies and instrumentation will surely improve the skills for the detection of pathogens.

## Figures and Tables

**Table 1:** Methods used in the area of microorganism identification.

Type	Basis	References
Indirect	- Conventional methods. Isolation and culture of microorganisms and the determination of their various phenotypic characteristics	[3]
Direct	- Culture-independent. May be used to identify specific microbes in a mixed population as well as identify non-culturable microbes. For example, microscopic techniques are powerful tools used in the identification of microorganisms by visualization of the characteristic structures and for organisms in the VBNC (viable but not culturable) state.	[4]
